# American Philosophical Society

**Published:** 1840-11

**Authors:** 


AMERICAN PHILOSOPHICAL SOCIETY.

Among the proceedings of this Society for May, June, and July, of the present year, a copy of which we have received, we find some interesting remarks on tornadoes, from which the following extracts are made:

"Dr. Hare adverted to the fact, that in an essay, published in Silliman's Journal in 1822, he had, agreeably to the authority of Dalton and Davy, stated, that the cold consequent on the rarefaction of air in its ascent towards the upper strata of the atmosphere was one of the causes of the formation of clouds; and in his text books he had soon after published an engraving of an apparatus, by means of which he was accustomed to illustrate, before his pupils the transient cloud which arises from a diminution of pressure in air containing aqueous vapour.

"In the essay above mentioned, Dr. Hare had alleged, that as much caloric was given out by aqueous vapour, during its conversion into snow, as would be yielded by twice the weight of red-hot powdered glass. But Mr. Espy, he considered, had the merit of being the first to suggest, that the heat, thus envolved, might be an important instrument in causing a buoyancy tending to accelerate any upward current of warm moist air.

"Dr. Hare had been willing to admit, that this transfer of heat might co-operate with other causes in the production of storms, but could not concur with Mr. Espy in considering it competent to give rise to thunder gusts, tornadoes or hurricanes. These he had considered, and still considers, to be mainly owing to electoral discharges between the earth and the sky; or between one mass of another."

"Professor Bache read an extract of a letter addressed by Mr. Forshey, of Natchez, to Mr. Espy, in reference to the tornado which occurred there recently.

"The writer stated that he had spent much time in examining the track of the storm in the vicinity of Natchez. He had ascertained its extent to have been not less than five or six miles below the city, and twenty miles beyond; its effects having been felt, but with less violence, for nearly one hundred and fifty miles. The track near Natchez was directed sixty degrees to the east of north. After describing the destruction of the city of Natchez, the writer states, that objects were every where blown towards the track of the storm; those directed most westwardly lying invariably below those directed more eastwardly. Mr. Forshey also describes the effect opon the houses as of an explosion outwards. In his view, these facts strongly confirm Mr. Espy's theory of this meteor."

"Mr. Walker made some remarks on the tornado, of limited extent, which visited Philadelphia on the 13th instant.

"Mr. Walker's own observations, and those of several intelligent individuals, on different sides of the central path, led him to the conclusion, that the currents from without the borders of the tornado were directed, in every instance, towards its centre. This was manifest from the motion of the clouds, in the different strata of the atmosphere. The theory of the central tendency of the currents in tornadoes, usually ascribed to Mr. Espy, was, Mr. Walker remarked, of older date, having been advanced by Franklin in the middle of the last century. The whirl, on which so much stress is laid by Mr. Redfield and Colonel Reid, was distinctly seen in the lower current, where the condensed vapor, resembling spent steam, moved round in a spiral, making several turns downwards, each of smaller dimensions than the preceding, and resembling the motion of water in a common whirlpool. This circumstance seemed, to Mr. Walker, somewhat contradictory to part of Mr. Redfield's theory, that of the gradual enlarge of the periphery of the whirl, whereas the motion in the present instance was in a spiral tending inwards." Y.

